# The Tumorigenic Properties of EZH2 are Mediated by MiR-26a in Uveal Melanoma

**DOI:** 10.3389/fmolb.2021.713542

**Published:** 2021-07-26

**Authors:** Yao Li, Mingmei Zhang, Huayin Feng, Shaya Mahati

**Affiliations:** ^1^ Department of Ophthalmology, Xinjiang Medical University Affiliated First Hospital, Urumqi, China; ^2^ Department of Oncology, Xinjiang Medical University Affiliated First Hospital, Urumqi, China

**Keywords:** microRNA-26a, EZH2, uveal melanoma, tumor progression, cell proliferation

## Abstract

**Background:** The polycomb group protein enhancer of zeste homolog 2 (EZH2) has been found to be highly expressed in various tumors, and microRNA-26a (miR-26a) is often unmodulated in cancers. However, the functions of these two molecules in uveal melanoma (UM) and their relationships have not been reported.

**Methods:** We explored the effects of the miR-26a–EZH2 axis in UM by examining the levels of miR-26a and EZH2. The EZH2 levels in various tumor types and the correlations between EZH2 levels and overall survival and disease-free survival were reanalyzed. The binding of miR-26a to the 3′-untranslated region of EZH2 mRNA was measured using the luciferase reporter assay. The regulation of EZH2 gene expression by miR-26a was also identified, and the effect of elevated EZH2 expression on UM cell function was further examined. Results: miR-26a was downregulated and EZH2 was upregulated in UM cells. Overexpression of miR-26a inhibited cell proliferation, and knockdown of EZH2 suppressed cell growth. EZH2 was a direct target of miR-26a in UM cells. The knockout of EZH2 mimicked the tumor inhibition of miR-26a in UM cells, whereas the reintroduction of EZH2 abolished this effect. In addition, a network of EZH2 and its interacting proteins (UBC, CDK1, HDAC1, SUZ12, EED) was found to participate in miR-26a-mediated tumor progression.

**Conclusion:** The newly identified miR-26a–EZH2 axis may be a potential target for the development of treatment strategies for UM.

## Introduction

Uveal melanoma (UM) is the most common primary intraocular malignancy in adults (Rodríguez et al., 2016). Both UM and cutaneous melanoma originate from melanocytes; however, UM is biologically and clinically different from other skin melanomas (van der Kooij et al., 2019). More than half of the main UMs are recurrent; no effective therapy exists for metastatic UM. Thus, studying the mutations associated with UM metastasis, proliferation, and survival may help researchers to understand the mechanisms of its etiology and metastasis, thereby facilitating the development of more effective therapies.

Micro-ribonucleic acids (miRNAs) are a class of small, noncoding, 21 to 25-nucleotide-long RNA molecules (Tomar et al., 2020). They can cause degradation or inhibit translation of target genes by targeting the 3′-untranslated region (3′-UTR) of mRNA. Thus, they can regulate cell proliferation, differentiation, and apoptosis (Lee et al., 2010; Matoulkova et al., 2012). A recent study found that the abnormal expression of miRNAs is closely related to tumors, and that it exerts an effect on tumor suppressor genes and proto-oncogenes *in vivo*, thus regulating the occurrence, development, and outcome of tumors (Iorio and Croce, 2012). Low levels of the micro-RNA miR-26a were found to be expressed in liver, lung, and prostate cancers, which were closely related to tumor recurrence, metastasis, and poor prognosis (Yang et al., 2013). However, reports on the regulatory role of miR-26a on UM are rare.

Enhancer of zeste homolog 2 (EZH2) is an essential component of the epitope, genetic polycomb repressive complex 2 (PRC2) (Zhang et al., 2017; Bugide et al., 2018). EZH2 is required to maintain the characteristics of cancer stem cells (CSCs), demonstrating the ability of multiple cancers to self-regenerate, and specific transcription patterns (C. Wang et al., 2012). It is also critical for achieving cellular motility, by actively regulating genes rich in cytoskeletal components that increase invasive cell populations and promote movement and metastasis of melanoma in the skin (Moore, 2013). Elevated EZH2 levels predicted a negative prognosis, higher risk of metastasis, and shorter survival in a cohort of 89 patients with UM (Cheng et al., 2017; Jin et al., 2020). Given the effectiveness of EZH2 in enhancing CSC dryness and motility, we hypothesized that EZH2 was delivered to tumor cells with enhanced malignant characteristics during UM liver metastasis.

In this study, we explored the effects of the miR-26a–EZH2 axis in UM. The levels of miR-26a and EZH2 were examined. The regulation of EZH2 gene expression by miR-26a was also identified. Increased levels of EZH2 reversed the inhibitory effect of miR-26a in UM. This study demonstrated an important role of the miR–26a-EZH2 axis in UM and illustrated its potential application in UM therapy.

## Materials and Methods

### Cell Culture

UM cell lines (92.1, Mel270, Omn2.3, and Omn1) and human choroid plexus epithelial cells (HCPEpiC) were provided by the ATCC (Manassas, VA, United States). All cells were cultured in high-glucose Dulbecco’s modified medium (Invitrogen, Gaithersburg, MD, United States) with 0.1 mg/ml streptomycin, 100 U/ml penicillin, and 10% (v/v) fetal bovine serum. Cell culture was carried out in a humidified cell incubator at a temperature of 37°C, with 5% CO_2_.

### Transfection

The 92.1 and Mel270 cells (5 × 10^5^ cells/well) were incubated overnight in 24-well plates. The miR-26a mimic and pcDNA3.1-EZH2 expression plasmid was synthesized at Genomics Pharmaceuticals (Shanghai, China). miR-26a mimics (50 nM), EZH2 plasmid (1 μg), and the corresponding negative control were transfected using lipofectamine 2000 (Invitrogen, Carlsbad, CA, United States) according to the manufacturer’s instructions. In some experiments, siRNA for EZH2 (si-EZH2) or its negative control were transfected into 92.1 and Mel270 cells. The sequences of si-EZH2 were as follows: 5′-GUC GCA ACG GAC CAG UUA A-3′. Scrambled siRNA (si-con) (5′-GGC GAU CAC GAC UAA GAC U-3′) served as a negative control.

### Cell Proliferation Assays

Cell proliferation was measured using 3-(4,5-dimethylthiazol-2-yl)-2,5-diphenyltetrazolium bromide (MTT) solution; Dojindo, Kumamoto, Japan). 92.1 and Mel270 cells (2000 cells/well) were seeded in 96-well plates for 5 more days, then reacted in a 10% MTT solution and incubated in a cell incubator (37°C, 5% CO_2_) for 2 h. Optical density values were measured at 570 nm.

### Luciferase Reporter Assays

The estimated binding site of miR-26a with wild-type (wt) and mutant (mut) EZH2 3′-UTR was cloned into the pmirGLO dual luciferase reporter vector (YouBio, Changsha, China). The reporter vectors (pre-miR-control, pre-miR-26a, and wt, and mut 3′-UTR EZH2) were coinfected into 92.1 and Mel270 cells. After 48 h, luciferase activity was detected using a standardized dual luciferase assay system (Promega, Madison, WI, United States).

### Quantitative RT-PCR

A TRIzol reagent (YouBio) was used to extract all RNA from the cell samples. Then, 2 μg of total RNA was reversed using the Script RT kit (YouBio). The ABI PRISM^®^ 7900 SYBR Green PCR Master Mixture (Applied Biosystems, Waltham, MA, United States) was used in the array detection system for quantitative reverse transcription polymerase chain reaction (qRT-PCR). GAPDH and U6 snRNA were used as reference genes, respectively. The primers used were as follows: miR-26a-5p-forward: 5ʹ-UCC AUA AAG UAG GAA ACA CUA CA-3ʹ; backward: 5ʹ-CAG UAC UUU UGU GUA GUA CAA-3ʹ. EZH2-forward: 5ʹ-CCC TGA CCT CTG TCT TAC TTG TGG A-3ʹ; backward: 5ʹ-ACG TCA GAT GGT GCC AGC AAT-3ʹ. GAPDH-forward: 5ʹ-GAA GGT GAA GGT CGG AGT C-3ʹ; backward: 5ʹ-GAA GAT GGT GAT GGG ATT TC-3ʹ. U6-forward: 5ʹ-CTC GCT TCG GCA GCA CAT ATA CT-3ʹ; backward: 5ʹ-ACG CTT CAC GAA TTT GCG TGT C-3ʹ. UBC-forward: 5ʹ-CTG GAA GAT GGT CGT ACC CTG-3ʹ; backward: 5ʹ-GGT CTT GCC AGT GAG TGT CT-3ʹ. CDK1-forward: 5ʹ-AAA CTA CAG GTC AAG TGG TAG CC-3ʹ; backward: 5ʹ-TCC TGC ATA AGC ACA TCC TGA-3ʹ. HDAC1-forward: 5ʹ-CTA CTA CGA CGG GGA TGT TGG-3ʹ; backward: 5ʹ-GAG TCA TGC GGA TTC GGT GAG-3ʹ. SUZ12-forward: 5ʹ-AGG CTG ACC ACG AGC TTT TC-3ʹ; backward: 5ʹ-GGT GCT ATG AGA TTC CGA GTT C-3ʹ. EED-forward: 5ʹ-GTG ACG AGA ACA GCA ATC CAG-3ʹ; backward: 5ʹ-TAT CAG GGC GTT CAG TGT TTG-3ʹ.

### Western Blotting

Cells were collected at a concentration of 30 μg/sample for sodium dodecyl sulphate–polyacrylamide gel electrophoresis (SDS-PAGE) using a pre-chilled radioimmunoprecipitation assay buffer cocktail with a phosphatase inhibitor. The polyvinylidene fluoride membrane (0.45 μm) was transferred to 1 × Tris-buffered saline solution with Tween-20 detergent (TBST) to block 2% bovine serum albumin. Then, the primary EZH2 antibody (#4905; Cell Signaling Technology) was cultured at 4°C (1:1,000) for 12 h, and the secondary antibody at 37°C for 1 h. After four rounds of TBST washing, the membrane was colored by chemiluminescence using the ECL Western Blotting Substrate Kit (ab65623; Abcam, Cambridge, MA, United States).

### Statistical Analyses

All experimental data were analyzed and plotted using GraphPad Prism 9 (GraphPad Software, San Diego, CA, United States). All data are presented as the mean ± standard error. Statistical differences between two groups were compared using the non-response Student t-test. One-way analysis of variance was used to analyze multiple groups. A *p*-value of <0.05 was considered statistically significant.

## Results

### Enhancer of Zeste Homolog 2 Was Upregulated and Promoted Tumor Proliferation in Uveal Melanoma

**FIGURE 1:**
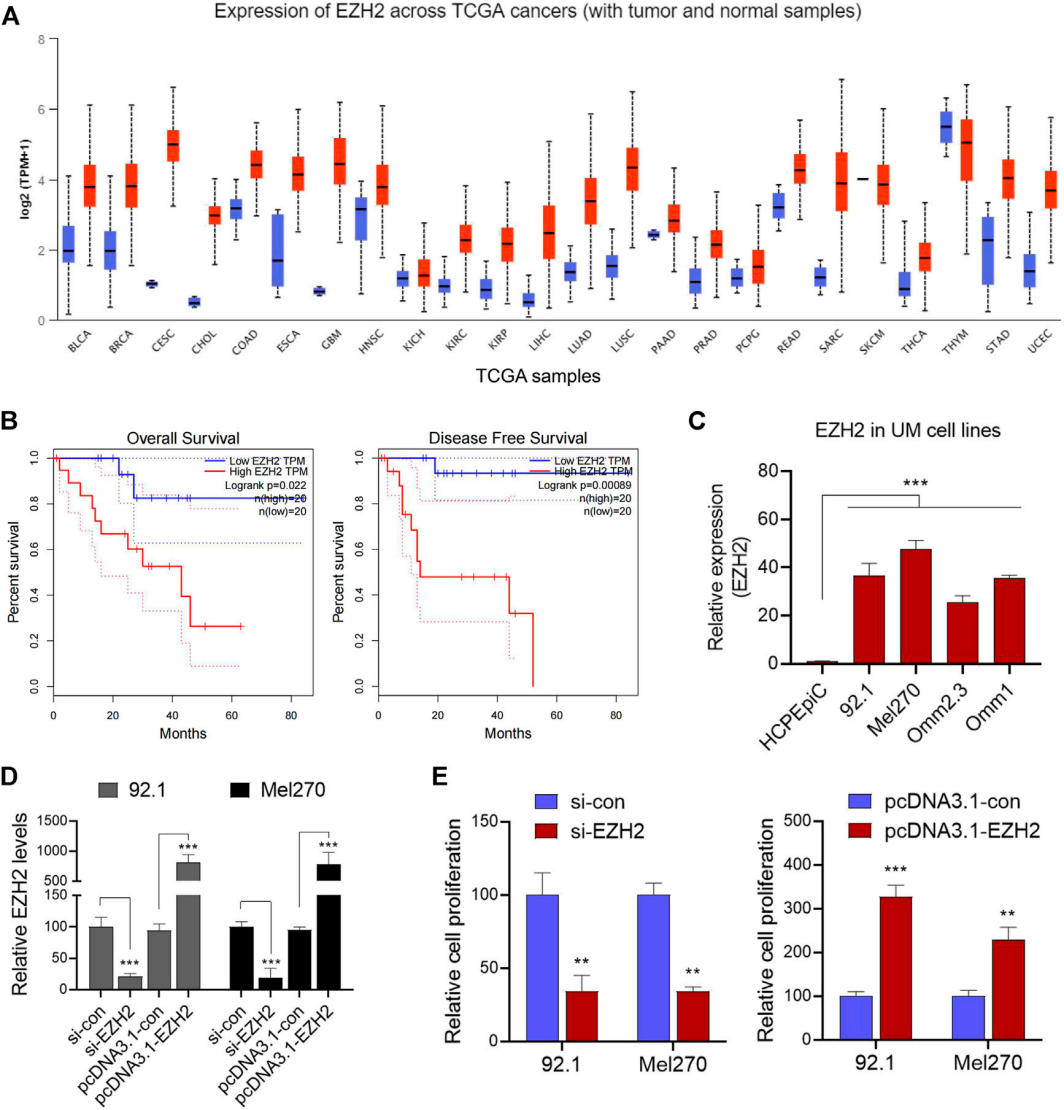
EZH2 was upregulated and promoted tumor proliferation in UM. **(A)** In various tumor types of the TCGA dataset, UALCAN analysis was used to reanalyze the levels of EZH2. **(B)** The correlations between the expression of EZH2 and overall survival (OS) and disease-free survival (DFS) were further analyzed in patients with UM. **(C)** Relative levels of EZH2 expression in the UM cell lines and a human choroid plexus epithelial cell line (HCPEpiC) were measured. **(D)** After the knockdown of EZH2 by siRNA, or overexpression of EZH2, relative EZH2 levels were determined in 92.1 and Mel270 cells. **(E)** Cell viabilities in both cell groups were evaluated. **p < 0.01, ***p < 0.001. Abbreviations: EZH2, enhancer of zeste homolog 2; UM, uveal melanoma; TGGA, The Cancer Genome Atlas.

We initially analyzed the EZH2 expression levels in various tumor types and corresponding normal tissues in the Cancer Genome Atlas dataset using UALCAN analysis (http://ualcan.path.uab.edu/analysis.html). The results revealed that EZH2 was upregulated in various tumor types (Figure 1A). Further analysis showed that high EZH2 expression was significantly associated with overall survival and disease-free survival in patients with UM (Figure 1B). Subsequently, EZH2 levels were found to be significantly higher in UM cells (92.1, Mel270, Omn2.3, and Omn1) than in normal HCPEpiC cells (*p* < 0.001, Figure 1C), suggesting that the upregulation of EZH2 might be associated with the development of UM. As expected, the knockdown of EZH2 by siRNA dramatically inhibited cell proliferation (Figures 1D,E); meanwhile, the overexpression of EZH2 significantly promoted the growth of tumor cells (Figures 1D,E).

### Enhancer of Zeste Homolog 2 Was Identified as a Target of microRNA-26a in Uveal Melanoma

**FIGURE 2:**
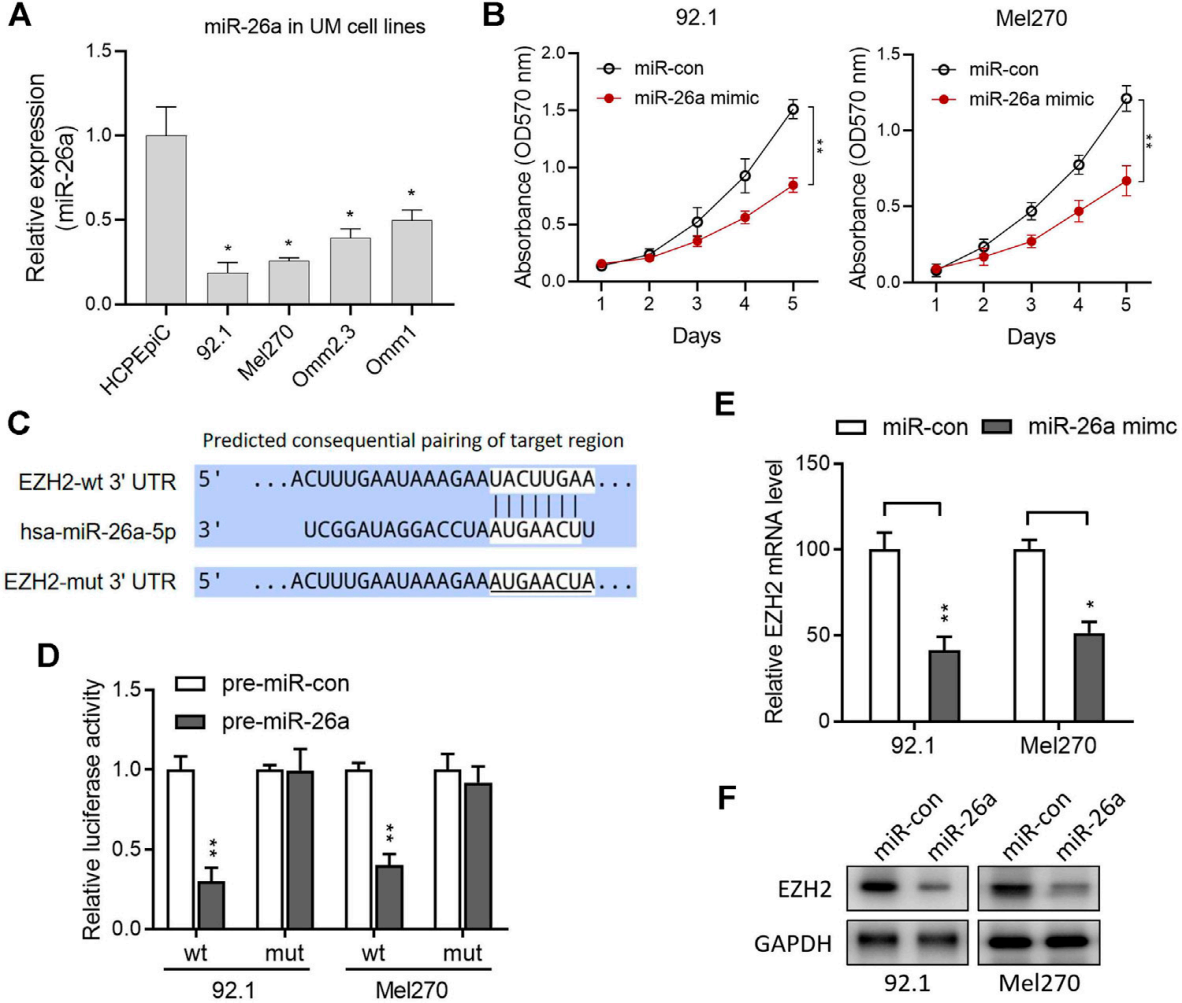
EZH2 was identified as a target of miR-26a in UM. **(A)** Relative levels of miR-26a expression in the UM cells. *p < 0.05 vs. HCPEpiC. **(B)** The effect of ectopic miR-26a expression on cell proliferation in the 92.1 and Mel270 cells was evaluated by the MTT assay. **(C)** The 3′-UTR of the EZH2 mRNA included the predicted binding domain of miR-26a. **(D)** The 92.1 and Mel270 cells were transfected with pre-miR-con or pre-miR-26a, and the reporter vectors with the full-length wild-type (wt) or mutant (mut) EZH2 3′-UTR; luciferase activity was detected. **(E)** qRT-PCR was used to evaluate the EZH2 mRNA levels in 92.1 and Mel270 cells with ectopic miR-26a expression (*p < 0.05, **p < 0.01 vs. miR-con). **(F)** The effects of ectopic miR-26a expression on the EZH2 protein levels of 92.1 and Mel270 cells were detected by western blotting. Abbreviations: UM, uveal melanoma; EZH2, enhancer of zeste homolog 2; HCPEpiC, human choroid plexus epithelial cell.

In UM cells, miR-26a expression was significantly lower than that in normal HCPEpiC cells (*p* < 0.050; Figure 2A). Furthermore, the miR-26a mimic significantly suppressed 92.1 and Mel270 cell proliferation, as evaluated by the MTT assay (Figure 2B; all *p* < 0.01). The candidate target genes of miR-26a were predicted using TargetScan (http://www.targetscan.org/) to elucidate the basic mechanism of miR-26a in UM. Potential miR-26a-binding sites in EZH2 3′-UTR were found (Figure 2C). A luciferase assay was used in 92.1 and Mel270 cells to confirm this prediction after transfection with wt or mut EZH2 luciferase reporter vectors in combination with pre-miR-26a or pre-miR-con. The overexpression of pre-miR-126a markedly reduced the luciferase activity of the wt reporter, rather than the mut reporter, in both cell types (Figure 2D). We performed qRT-PCR and western blotting to confirm this finding. The results revealed that miR-26a overexpression with the mimic significantly reduced EZH2 mRNA and protein levels in 92.1 and Mel270 cells (Figures 2E,F).

### microRNA-26a Suppressed Tumor Progression by Targeting Enhancer of Zeste Homolog 2

**FIGURE 3:**
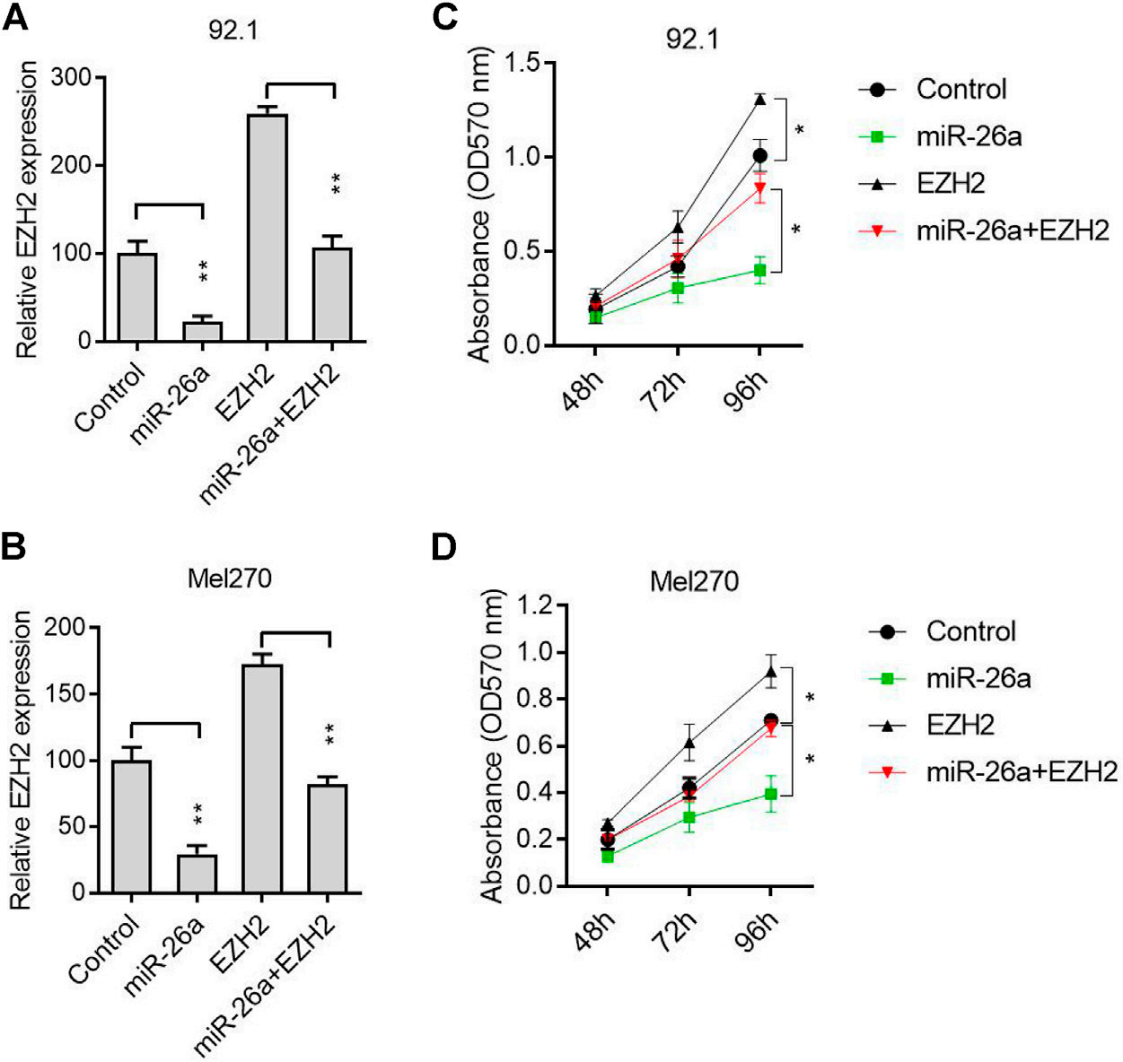
miR-26a suppressed tumor progression by targeting EZH2. **(A)** After transfection with pcDNA3.1-EZH2 and/or miR-26a mimic in the 92.1 cells, EZH2 expression was evaluated using qRT-PCR (**p < 0.01). **(B)** qRT-PCR analysis was performed to evaluate EZH2 expression in Mel270 cells (**p < 0.01). **(C)** MTT assays were performed in 92.1 cells (*p < 0.05). **(D)** Cell proliferation was assessed in Mel270 cells (*p < 0.05).

A rescue experiment was conducted to further verify that EZH2, as a target gene, participated in the antitumor process induced by miR-26a. The 92.1 and Mel270 cells were transfected with a miR-26a mimic or EZH2 expression vector. The EZH2 mRNA level was inhibited by the miR-26a mimic, and reversed by the EZH2 expression vector, in 92.1 (Figure 3A) and Mel270 cells (Figure 3B). Furthermore, EZH2 overexpression significantly abolished the inhibition of 92.1 and Mel270 cell proliferation induced by miR-26a (Figures 3C,D).

### A Network of Enhancer of Zeste Homolog 2 and its Interacting Proteins Participated in microRNA-26a-Mediated Tumor Progression

**FIGURE 4:**
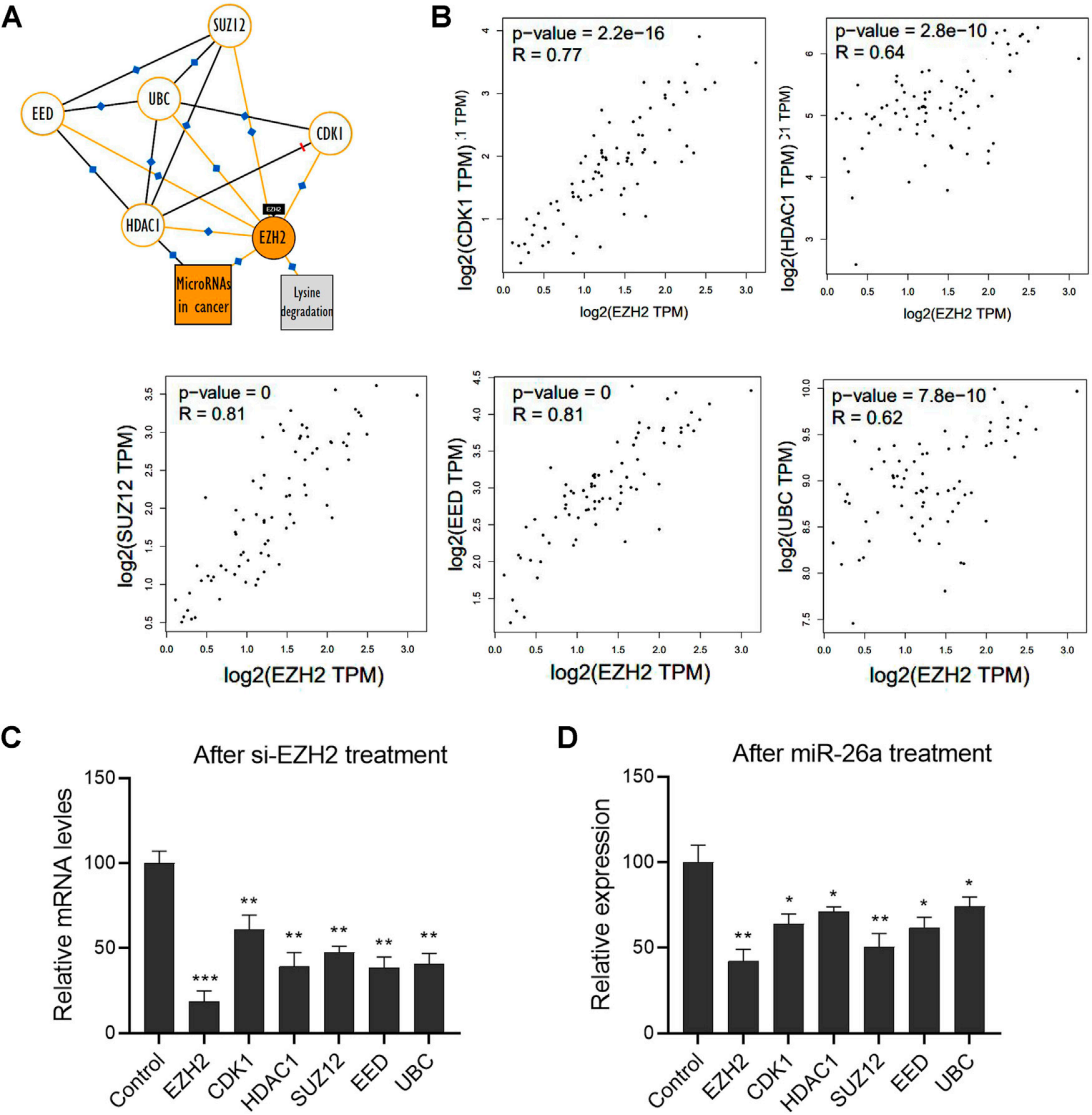
A network of EZH2 and its interacting proteins participated in miR-26a-mediated tumor progression. **(A)** The Kyoto Encyclopedia of Genes and Genomes pathway analysis was performed to identify potential interacting partners of EZH2 in UM. **(B)** The interaction between EZH2 and these molecules was analyzed using the non-log scale for calculation and log-scale axis for visualization (Pearson’s correlation coefficient). **(C)** qRT-PCR analysis was performed to evaluate mRNA expression in 92.1 cells after EZH2 gene silencing. **(D)** After transfection with a miR-26a mimic, mRNA levels of EZH2, UBC, CDK1, HDAC1, SUZ12, and EED were measured in 92.1 cells (*p < 0.05, **p < 0.01, ***p < 0.001).

A Kyoto Encyclopedia of Genes and Genomes pathway analysis was performed to identify the potential interacting partners of EZH2 and describe the possible signaling pathways in UM. We found a panel of molecules involved in the EZH2-mediated signaling pathway, including UBC, CDK1, HDAC1, SUZ12, and EED (Figure 4A). We further analyzed the interaction between EZH2 and these molecules, using the non-log scale for calculation and the log-scale axis for visualization. Pearson correlation coefficients revealed significant positive correlations with EZH2 (Figure 4B). Indeed, EZH2 gene silencing significantly downregulated the mRNA expression levels of these molecules (Figure 4C). Notably, the results show that miRNAs may also be involved in regulating the EZH2-mediated signal network. After transfection with a miR-26a mimic, the mRNA levels of these molecules, including EZH2, UBC, CDK1, HDAC1, SUZ12, EED, were significantly downregulated in 92.1 cells (Figure 4D).

## Discussion

Most patients with UM do not seek treatment until blurred vision, metamorphopsia, or visual hallucination occurs; hence, they tend to miss the best opportunity for treatment (Yang et al., 2013). Studies have shown that miRNA expression varies at different stages of UM, suggesting that miRNAs are involved in regulating the occurrence, development, and invasion of UM (Yang et al., 2013). The involvement of miR-26a in the regulation of tumor cell proliferation, metastasis, and apoptosis has been well documented in multiple studies (Jia et al., 2014; Shao et al., 2016; Cai et al., 2017). In this study, we found that the miR-26a–EZH2 axis was involved in the occurrence and development of UM, by downregulating the expression of miR-26a and upregulating the expression of EZH2.

Initially, it was determined that miR-26a was underexpressed and EZH2 was highly expressed in UM cell lines (Bande et al., 2020). EZH2, a catalytic subunit of PRC2, is an oncogenic protein that silences the expression of various tumor-inhibitor miRNAs, such as miR-125b, miR-139-5p, miR-101, let-7c, and miR200b, and is typically increased in human cancers (Wang et al., 2016; Ma et al., 2018). Interestingly, previous studies have shown that EZH2 overexpression promotes growth within UM (Jin et al., 2020; Wu et al., 2020). The absence of EZH2 or the upregulation of miR-26a significantly impaired the proliferative capacity of UM cells in this study.

The function of miRNA depends mainly on the target gene (Mukherji et al., 2011). EZH2 plays a key role in tumor formation via epigenetic gene silencing and chromatin remodeling (Yamagishi and Uchimaru, 2017). miR-25, −26a, −30 days, -98, and let-7 interact with the defined sequences in EZH2 3′-UTR to directly diminish EZH2-regulated proteins (Zhu et al., 2016). To investigate potential mechanisms involving miR-26a and EZH2, we predicted potential miR-26a-binding sites in the 3′-UTR of EZH2 using the TargetScan database. EZH2 was further identified as a target gene of miR-26a using the luciferase assay, qRT-PCR, and western blotting.

The overexpression of miR-26a suppressed the growth of UM cells by promoting the apoptosis of UM cells, which has been reported to be related to the regulation of the p53/MDM2 pathway (Krell et al., 2013). EZH2 inhibitors suppressed the growth of UM cells by adjusting lysine methylation activity, thereby making EZH2 an effective therapeutic target (Ler et al., 2017; Jin et al., 2020). However, the regulatory relationship between EZH2 and miR-26a in UM tumors has not been reported. We demonstrated that miR-26a inhibited cell survival by directly targeting EZH2, and that EZH2 overexpression reversed the miR-26a-dependent inhibiting effects. The potential interacting partners of EZH2 (including UBC, CDK1, HDAC1, SUZ12, EED) were predicted and identified, which constituted the PRC2. This contributed to tumorigenesis by inducing the epigenetic silencing of gene expression (Baylin and Ohm, 2006). In subsequent studies, we will further explore the effect of the miR-26a–EZH2 axis *in vivo* and evaluate the potential application value of EZH2 inhibitors and miRNA drugs in UM.

## Conclusion

This study showed that miR-126a acts as a tumor inhibitor in UM and suppresses cell viability by targeting the EZH2 gene. Therefore, the miR-26a–EZH2 axis may be a potential target for UM therapy.

## Data Availability

The original contributions presented in the study are included in the article/supplementary material, further inquiries can be directed to the corresponding author.
